# Effect of alfalfa supplementary change dietary non-fibrous carbohydrate (NFC) to neutral detergent fiber (NDF) ratio on rumen fermentation and microbial function in Gansu alpine fine wool sheep (*Ovis aries*)

**DOI:** 10.1080/10495398.2023.2262539

**Published:** 2023-10-02

**Authors:** Qian Chen, Yun-feng Cui, Zhao-xi Zhang, Fu-cheng Jiang, Xiang-yu Meng, Jin-jin Li, Da-yong Cui, Jian-lei Jia

**Affiliations:** a College of Agriculture and Animal Husbandry, Qinghai University, Xining, Qinghai, P.R. China; b School of Life Science, Qilu Normal University, Jinan, Shandong, P.R. China; c The Bureau of Animal Industry of Zhangqiu, Jinan, Shandong, P.R. China; d Canada Lallemand Inc, Beijing Office, Beijing, P.R. China

**Keywords:** Gansu alpine fine-wool sheep, alfalfa supplementary, nfc/ndf, rumen fermentation, microbial function

## Abstract

Bodyweight loss and rumen microbial dysfunction of grazing sheep was a challenge for the sheep production industry during cold season, which were considered to correlated with under-roughage-feeding. Alfalfa is a good roughage supplementary for ruminants, which can improve grazing sheep bodyweight-loss and rumen microbial dysfunction during grass-withering period. This study evaluated the effects of alfalfa hay supplementary change dietary non-fibrous carbohydrate/neutral detergent fiber (NFC/NDF) ratios on rumen fermentation and microbial function of Gansu alpine fine wool sheep during extreme cold season. 120 ewes (3–4 yrs) with an average body weight of 28.71 ± 1.22 kg were allocated randomly into three treatments, and fed NFC/NDF of 1.92 (H group), 1.11 (M group), and 0.68 (L group), respectively. This study was conducted for 107 d, including 7 d of adaption to the diets. The rumen fermentation parameters and microbial characteristics were measured after the end of feeding trials. The results showed that the concentrations of sheep body weight, nitrogen components (Total-N, Soluble protein-N and Ammonia-N), blood biochemical indices (LDH, BUN and CHO) and ruminal volatile fatty acids (TVFA and propionate) significantly increased with an increase in the proportion of NFC/NDF ratios (*p* < .05), and the acetate and acetate/propionat ratio presented a contrary decreasing trend (*p* < .05). A total of 1018 OTUs were obtained with 97% consistency. Ruminococcus, Ruminococcaceae and Prevotella were observed as the predominant phyla in ruminal fluid microbiota. Higher NFC/NDF ratios with Alfalfa supplementary increased the richness and diversity of ruminal fluid microbiota, and decreased ruminal fluid microbiota beta-diversity. Using clusters of orthologous groups (COG), the ruminal fluid microbiota of alfalfa supplementary feeding showed low immune pathway and high carbohydrate metabolism pathway. In summary, the study suggested that there was an increasing tendency in dietary NFC/NDF ratio of 1.92 in body weight, ruminal fermentation, microbial community composition and fermentation characteristics through developing alfalfa supplementary system.

## Introduction

The Gansu alpine fine-wool sheep (GS) is the first cultivated breed of plateau type fine wool-sheep in Sunan plateau area, which could well-adapted to The Sunan plateau (Zhangye, Gansu) harsh environmental conditions, such as high altitude, low air oxygen and short forage growing season.^1^ The GS grazed grassland all year round, and highly dependent on pasture resources, however, long-lasting dormant season and decline in nutritive value of pasture making it difficult to meet nutritional requirement for sheep, resulting in growth retardation, hypoimmunity and higher mortality rate.^2^ Previous researches demonstrated that there are positive effects of hay supplementary on sheep productive and reproductive performance, which can reduce body weight-lost, feeding costs, and increase economic efficiency.^3^ In order to reduce the pressure of grazing on natural pastures and solve the inherent contradiction between production and ecology, a feasible breeding strategy for the transition from traditional farming to modern farming is essential in pasture-based animal husbandry. It follows that there is a big potential to improve animal performance through developing compensatory feeding system in dormant season.

Alfalfa has the advantages of rich nutrition and high quality, which is great significance to promote animal health and improve animal production performance.^4^ Alfalfa was widely planted in Gansu Province, and had been well received in animal husbandry as high-quality roughage.^5^ Many studies show that adding appropriate alfalfa hay to sheep roughage could improve diet digestion, however, with the elevated proportion, the digestibility has a downward trend, which was resulting in a negative effect.^6^^,^^7^ Dietary interventions can improve production traits via changing gastrointestinal microflora abundance.^8^ The composition of microbial communities of the ruminants was highly responsive to changes in diet structure, thereby affecting the efficiency of microbial fermentation and ruminal function.^9^

Rumen was an important organ of nutrition digestion, absorption and metabolism, also a main barrier against harmful substances.^10^ The rumen is colonized by diverse microbes, including bacteria, fungi, and protozoa,^11^ which enable the host to utilize the ingested feed and play an important role in the digestion and absorption of nutrients.^12^^,^^13^ The adjustment of the ratio of non-fibrous carbohydrate to neutral detergent fiber (NFC/NDF) alters ruminal fermentation and microbial type in ruminants.^14^ The composition of microbial communities of the ruminants was highly responsive to changes in diet structure, thereby affecting the efficiency of microbial fermentation and ruminal function.^15^ The compositions of microbial communities of the ruminants were active response to change in diets composition, physiological status, and feeding pattern.^16^ Dietary protein and energy are often concomitantly limited in grazing sheep during dormant season, some studies showed that sheep could exhibit high dry matter, fiber digestion and efficient protein utilization when limiting by energy intake.^17^ Therefore, an appropriate diet structure is essential for the healthy growth of Gansu alpine fine-wool sheep and economic benefits. The objective of the present study was to examine the effect of dietary percentage of NFC/NDF ratio on rumen fermentation and microbial function in Gansu alpine fine wool sheep, which may provide a theoretical basis for the formulation of feeding and management of Gansu alpine fine wool sheep.

## Materials and methods

### Ethics statement

This study was approved by the Institutional Animal Care and Use Committee of Qinghai University (Protocol 20170407-6). All research involving animals (sheep feeding, blood sample collection, rumen fluid collection) was conducted according to either the Guide for the Care and Use of Laboratory Animals (8th edition, National Academies Press).

### Experiment design

The study was carried out in Yugur Autonomous County of Sunan, Gansu Province, China, which is situated at Qilian Mountain of northeastern Qinghai-Tibetan Plateau. This area is over 3000 m above the sea level and has a dry cold climate.^18^

A total of 120 Gansu alpine fine wool ewes (aged 3 to 4-yr-old and 28.71 ± 1.22 kg), which were housed at the Sunan herdsmen’s professional cooperatives (Zhangye state, Gansu province, China), were allotted randomly into three groups (40 ewes/group). Four dietary treatments were formulated to produce different NFC/NDF ratios (Table 1), including 1.92 (H group), 1.11 (M group), and 0.68 (L group), respectively. The experiment lasted for 100 d from 16th November 2017 to 23rd March 2018, where the first 10 d was adjustment period, followed by 90 d of data collection period. All individuals were fed twice daily at 8.30 and 16.30 and given free access to diets and water.

**Table 1.** Dietary formula and proximate nutrition content.

Items	Groups		
	H	M	L
Ingredients (%)			
alfalfa hay	15	25	35
Alfalfa silage	15	25	35
Corn	40.15	30.46	16.28
Rapeseed meal	14.23	7.25	2.34
Cottonseed meal	6.29	3.25	2.12
Soybean meal	2.33	2.04	2.26
NaCl	0.5	0.5	0.5
Limestone	0.5	0.5	0.5
NaHCO3	0.5	0.5	0.5
CaHPO4	0.5	0.5	0.5
Premix^a^	5	5	5
Total	100	100	100
Nutrient levels^b^			
DE, MCal/kg	3.022	3.013	3.005
Crude protein, %	13.88	13.82	13.79
Ash, %	4.66	4.64	4.65
Ether extract, %	4.01	4.91	4.97
Neutral detergent fiber (NDF), %	26.53	36.29	45.54
Non-fiber carbohydrates (NFC), %	50.92	40.34	31.05
NFC/NDF^c^	1.92	1.11	0.68
Ca, %	1.12	1.12	1.12
P, %	0.23	0.24	0.24

^a^ Provided per kilogram of diets: Cu 15 mg, Fe 55 mg, Zn 25 mg, Mn40 mg, Se 0.30 mg, I 0.5 mg, Co 0.20 mg, VA 20,000 IU, VD 4000 IU, VE 40 IU.

^b^ ME and NFC were calculated values according to the formula in Nutritional Requirements of Sheep for Meat in China, while other nutrient levels were measured values.

^c^ NFC was calculated according to the following equation: NFC = 100 − (% CP + %NDF + %EE + %mineral matter) (Mertens, 1997).

### Sample collection

The sheep were weighed at 1 day and 90 days of sample collection period by using platform scale before feeding in the morning.

Jugular blood (10 mL) was collected into heparinized vacutainer tubes and centrifuged at 2500 rpm for 15 min. Plasma samples were stored at −20 °C for analysis.

The stomach tube was inserted into the rumen to collect rumen fluid. Approximately 50–100 ml of rumen fluid was collected from each sheep, strained through 4 layers of cheesecloth and then the pH was determined immediately.

### Blood biochemical indices and rumen fermentation analysis

Total protein (TP), Albumin (Alb), alanine aminotransferase (ALT), Aspartate aminotransferase (AST), triglyceride (TG), cholesterol (CHO), glucose (GLU), lactate dehydrogenase (LDH) was measured colorimetrically with commercially TaKaRa kits.

The rumen fluid was divided into two parts. One part of rumen fluid samples was aliquoted into 10 mL sterile tubes and analyzed ruminal fermentation. Ruminal TVFA, including acetic acid, propionic acid, butyric acid, analyzed by gas chromatography (TRACE GC 1300, Thermo Scientific, USA) using a capillary column (AT-FFAP: 30 m). Ammonia-N, soluble protein-N and urea-N were determined by colorimetry (GENESYS 10S Vis, Thermo Scientific, USA).^19^

Another part of rumen fluid sampling was analyzed ruminal microbial characteristics. 16S rRNA gene sequencing detection was outsourced to BIOMARKER (Beijing, China). Total bacteria of rumen fluid sample were utilized for RNA extraction. Illumina HiSeq 2500 sequencing of 16S rRNA gene was performed to characterize microbial diversity and community composition. The V3-V4 hypervariable region of the microbial 16S rRNA gene were amplified by PCR according to primers forward (5′-CCTACGGGNGGCWGCAG) and reverse (5′-GGACTACHVGGGTATCTAAT).^20^ The cycling protocol was 95 °C for 2 min; 35 cycles at 95 °C for 2 min; 72 °C for 30 s; and 72 °C for 5 min. The 16S rRNA analysis was performed by R software (version 3.1.2), QIIME software (version 1.9.1) and UPARSE software. According to the UPARSE pipeline, multiplexed reads were clustered into operational taxonomic units (OTUs) based on 97% sequence identity. The 16S rRNA gene sequence was classified by RDP Classifier (version 2.2).

### Statistical analysis

The data were analyzed using a randomized complete block design using Statistics Analysis System (SAS) software version 19.0 (SAS Inc.; North Carolina, USA).^21^ The data analysis method adopted in the ANOVA was the Least Significant Ranges method (LSR-SSR). The relationship between fermentation parameters and rumen microbes of Gansu alpine fine-wool sheep was analyzed by Pearson’s correlation coefficient.^22^ The results of the analysis were given as mean ± SE. Differences were considered as significant at *p* < .05 and as a trend at *p* < .1.

## Results

### Dietary NFC/NDF with body weight

Table 2 presents the effect of different dietary NFC/NDF ratios on initial weights, final weights and daily body weight gain of Gansu alpine fine wool sheep. The final weights and daily body weight gain of H group and M group were significantly higher than L group (*p <* .05), and there was an increasing tendency of L group in daily body weight gain (*p <* .05). The results showed that the final weights and daily body weight gain significantly increased with an increase in the proportion of NFC/NDF ratios (*p* *<* .05).

**Table 2.** Effect of dietary non-fibrous carbohydrate (NFC) to neutral detergent fiber (NDF) ratio with body weight.

Items	H (1.92)	M (1.11)	L (0.68)
Initial Weight (Kg)	27.97 ± 0.95	28.31 ± 0.45	28.03 ± 0.72
Final Weight (Kg)	35.08 ± 0.84b	34.67 ± 0.64b	33.57 ± 0.77a
Average daily gain (g/d)	78.89 ± 4.03a	70.37 ± 3.25b	61.56 ± 4.43c

### Dietary NFC/NDF with blood biochemical indexes

Table 3 presents the effect of different dietary NFC/NDF ratios on blood biochemical indexes of Gansu alpine fine wool sheep. The BUN Value was above normal range, and others were within the normal range. The LDH, BUN and CHO value of H group and M group were significantly greater than L group (*p < .05*), and there were no significant differences in TP, Alb, AST, ALT, TG and GLU value among all groups (*p > .05*).

**Table 3.** Effect of dietary non-fibrous carbohydrate (NFC) to neutral detergent fiber (NDF) ratio with Blood biochemical indices.

Items	H (1.92)	M (1.11)	L (0.68)	Normal range
TP g/L	65.67 ± 1.55	62.25 ± 0.75	63.28 ± 1.18	47.30–78.03
Alb g/L	32.75 ± 0.48	32.33 ± 0.33	32.42 ± 0.51	15.48–38.11
AST U/L	133.48 ± 3.08	130.25 ± 4.31	129.81 ± 4.11	106.5–142.5
ALT U/L	30.85 ± 2.14	30.25 ± 1.85	30.18 ± 1.59	19.5–34.8
BUN mmol/L ↑	7.45 ± 0.06a	7.21 ± 0.15b	7.25 ± 0.17b	2.9–7.3
TG mmol/L	0.26 ± 0.01	0.23 ± 0.02	0.02 ± 0.01	0.14–1.09
CHO mmol/L	2.93 ± 0.04a	2.62 ± 0.02b	2.54 ± 0.06b	2.0–3.4
LDH mmol/L	303.00 ± 9.51a	286.00 ± 5.72b	289.37 ± 7.98b	233.3–341.5
GLU mmol/L	2.31 ± 0.22	2.22 ± 0.27	2.31 ± 0.17	1.5–2.5

### Dietary NFC/NDF with rumen fermentation parameters

Table 4 presents the effect of different dietary NFC/NDF ratios on rumen fermentation parameters of Gansu alpine fine wool sheep. Total-N and Soluble protein-N of H group and L group were significantly higher than that of M group (*p <* .05), Ammonia-N of H group were significantly greater than M group and L group (*p <* .05), there was no significant difference in ruminal pH and Urea-N among all groups (*p* > .05). The ruminal Volatile Fatty Acids (VFAs) of different dietary non-fibrous carbohydrate (NFC) to neutral detergent fiber (NDF) ratio were listed in Table 5, The concentrations of TVFA and propionate significantly increased with an increase in the proportion of NFC/NDF ratios (*p* < .05). The acetate and acetate/propionat ratio presented a contrary decreasing trend with an increase in dietary alfalfa concentration (*p* < .05).

**Table 4.** Effect of dietary non-fibrous carbohydrate (NFC) to neutral detergent fiber (NDF) ratio with rumen fermentation parameters.

Items	pH	Ammonia-N	Urea-N	Total-N	Soluble protein-N
H (1.92)	6.44 ± 0.11	24.24 ± 0.70a	1.15 ± 0.04	134.29 ± 0.56a	112.65 ± 0.92a
M (1.11)	6.41 ± 0.07	20.79 ± 0.15b	1.09 ± 0.05	125.31 ± 0.12b	102.40 ± 0.05b
L (0.68)	6.43 ± 0.58	21.23 ± 0.71b	1.11 ± 0.03	129.54 ± 1.23a	106.37 ± 0.73a

**Table 5.** Effect of dietary NFC/NDF with ruminal VFAs.

Items	TVFA(mmol/L)	Acetate(%)	Propionate(%)	Butyrate(%)	Acetate/ Propionate
H (1.92)	59.49 ± 3.28a	27.23 ± 4.03	21.39 ± 0.91a	7.34 ± 0.78a	1.28 ± 0.12c
M (1.11)	55.97 ± 1.96a	28.46 ± 3.15	17.29 ± 0.54b	7.64 ± 0.38a	1.64 ± 0.13b
L (0.68)	48.71 ± 3.16b	29.90 ± 2.49	12.93 ± 0.62c	4.80 ± 0.26b	2.32 ± 0.08a

### Dietary NFC/NDF with rumen microbial community and function

**Figure 1.**
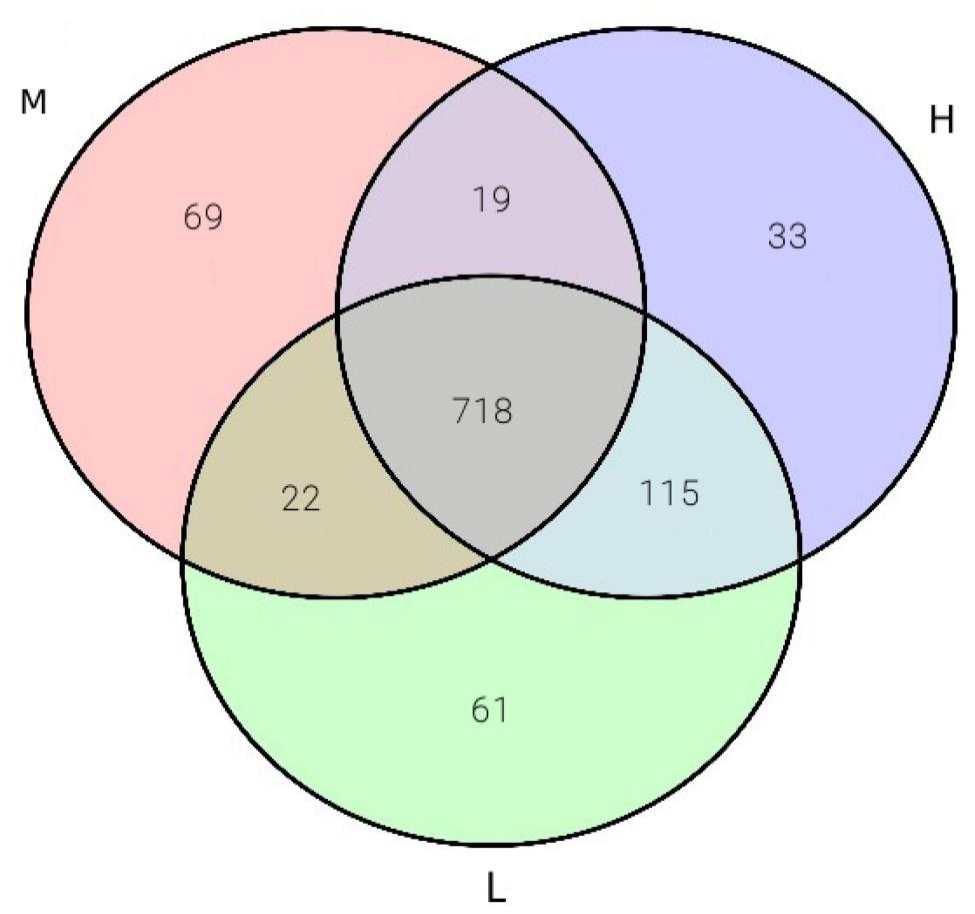
Ruminal fluid samples microbiota venn diagram.

**Figure 2.**
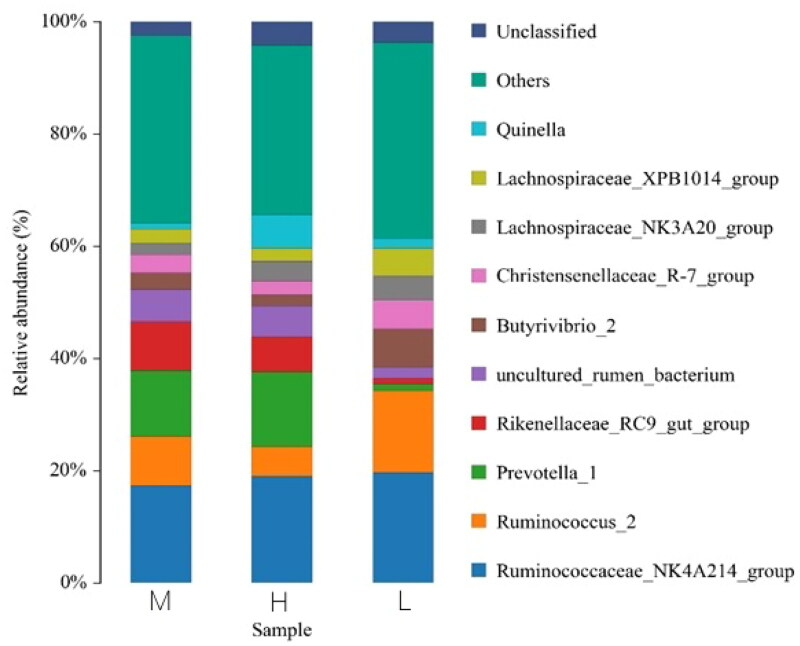
Percent of community abundance of microorganism.

After filtering out low-quality reads and chimeras of Illumina sequenced reads, we obtained a total of 987,146 clean tags high-quality sequences. The number of sequences for 18 sample ranged from 8784,781 to 985,217. The Good’s coverage was in the range of 0.9991–0.9998. The remaining high-quality sequences were clustered into OTUs according to 97% similarity level by UPARSE software. All detected OTUs were 24,148 and the averaged value of OTUs for each sample was 1136, which mapped to 21 phyla, 41 classes, 89 orders, 147 families, 297 genera and 324 species (Fig. 1). The top 10 relative ruminal fluid microbiota abundances at the genus level are presented in Figure 2. Ruminococcus, Ruminococcaceae and Prevotella were observed as the predominant phyla in ruminal fluid microbiota, followed by Christensenellaceae, Quinella, Rikenellaceae and Butyrivibrio. The relative abundances of Ruminococcus, Christensenellaceae, Ruminococcaceae and Prevotella accounted for more than 1% of the total microbiome. For A group, the relative abundance of beneficial microorganisms was significantly increased (*p* < .05) compared with other groups (*p* < .05). Ruminococcus and Quinella of A group and B group were significantly greater than C group and D group (*p <* .05).

**Figure 3.**
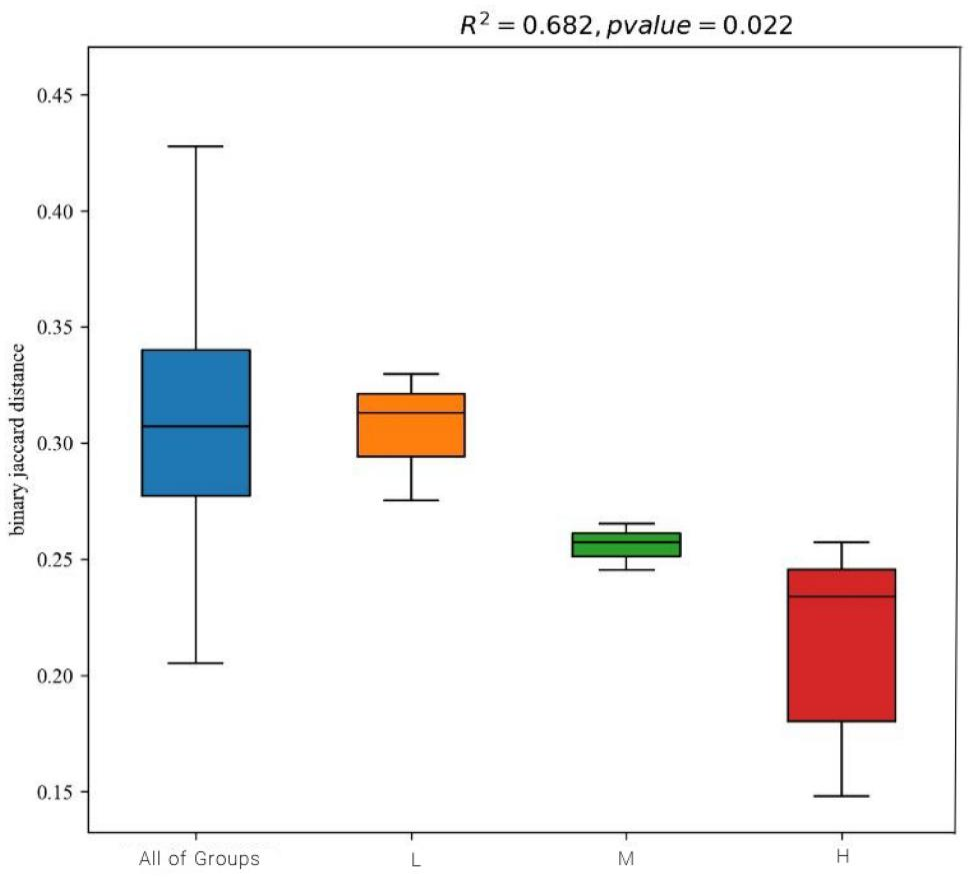
Ruminal fluid samples microbiota beta-diversity analysis.

**Figure 4.**
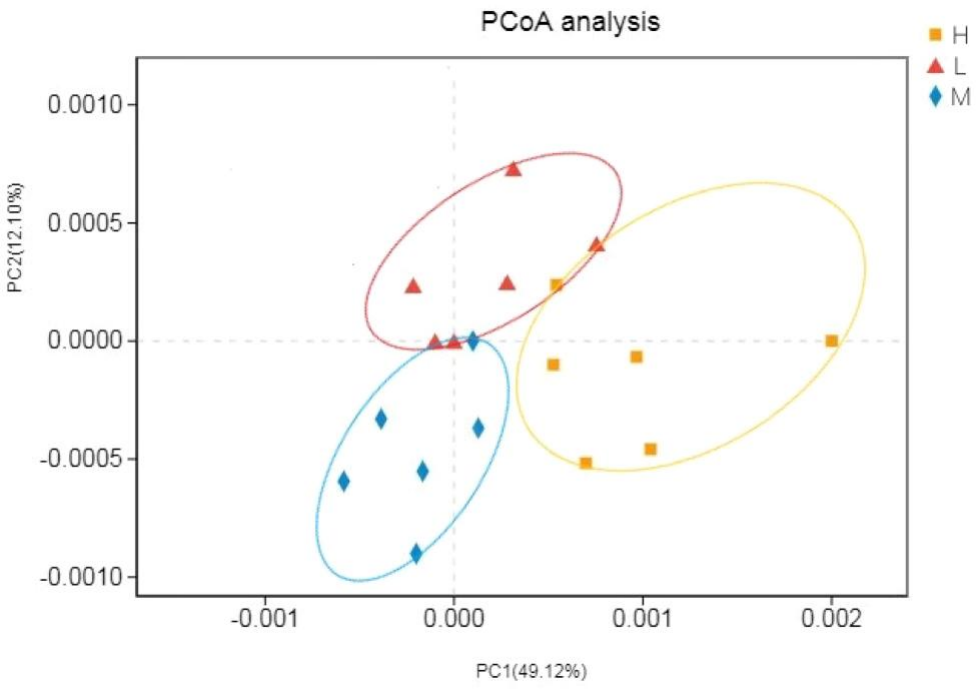
Ruminal fluid samples microbiota PCoA analysis.

The alpha diversity was estimated through the diversity index (Shannon and Simpson) and richness estimate (Chao1 and ACE). As can be seen in Table 6, the richness estimate (Simpson, ACE and Chao1) increased significantly for H group and M group compared to L group, whereas the Shannon indices were significantly lower than L group (*p* < .05). H group almost showed significant enhancement in the alpha-diversity of ruminal fluid microflora than other groups, this indicated that 1.92 of dietary non-fibrous carbohydrate (NFC) to neutral detergent fiber (NDF) ratio increased the richness and diversity of ruminal fluid microbiota. Furthermore, the differences in the microbial beta-diversity between the different feeding groups were evaluated based on binary jaccard distances (Fig. 3). The H group and M group showed lower ruminal fluid microbiota beta-diversity, which indicated their higher microbiota stability and lower dispersion. Principal co-ordinates analysis (PCoA) using the weighted unifrac similarity method revealed that the PC1 and PC2 explained 49.12% and 12.10% of the variation between the samples, respectively, it indicated that ruminal fluid samples from different groups formed different clusters in the ordination space (Fig. 4).

**Table 6.** Effect of dietary non-fibrous carbohydrate (NFC) to neutral detergent fiber (NDF) ratio with ruminal fluid samples alpha diversity.

Items	A (1.92)	C (1.11)	D (0.68)
Shannon	4.73 ± 0.21a	4.13 ± 0.28b	4.71 ± 0.29a
Simpson	0.027 ± 0.08b	0.064 ± 0.019a	0.032 ± 0.007b
ACE	758.33 ± 9.99b	878.87 ± 3.47a	747.25 ± 5.35b
Chao1	702.43 ± 17.47b	873.69 ± 15.07a	747.99 ± 16.33b
Coverage/%	98.33	98.67	98.36

### Dietary NFC/NDF with function prediction

**Figure 5.**
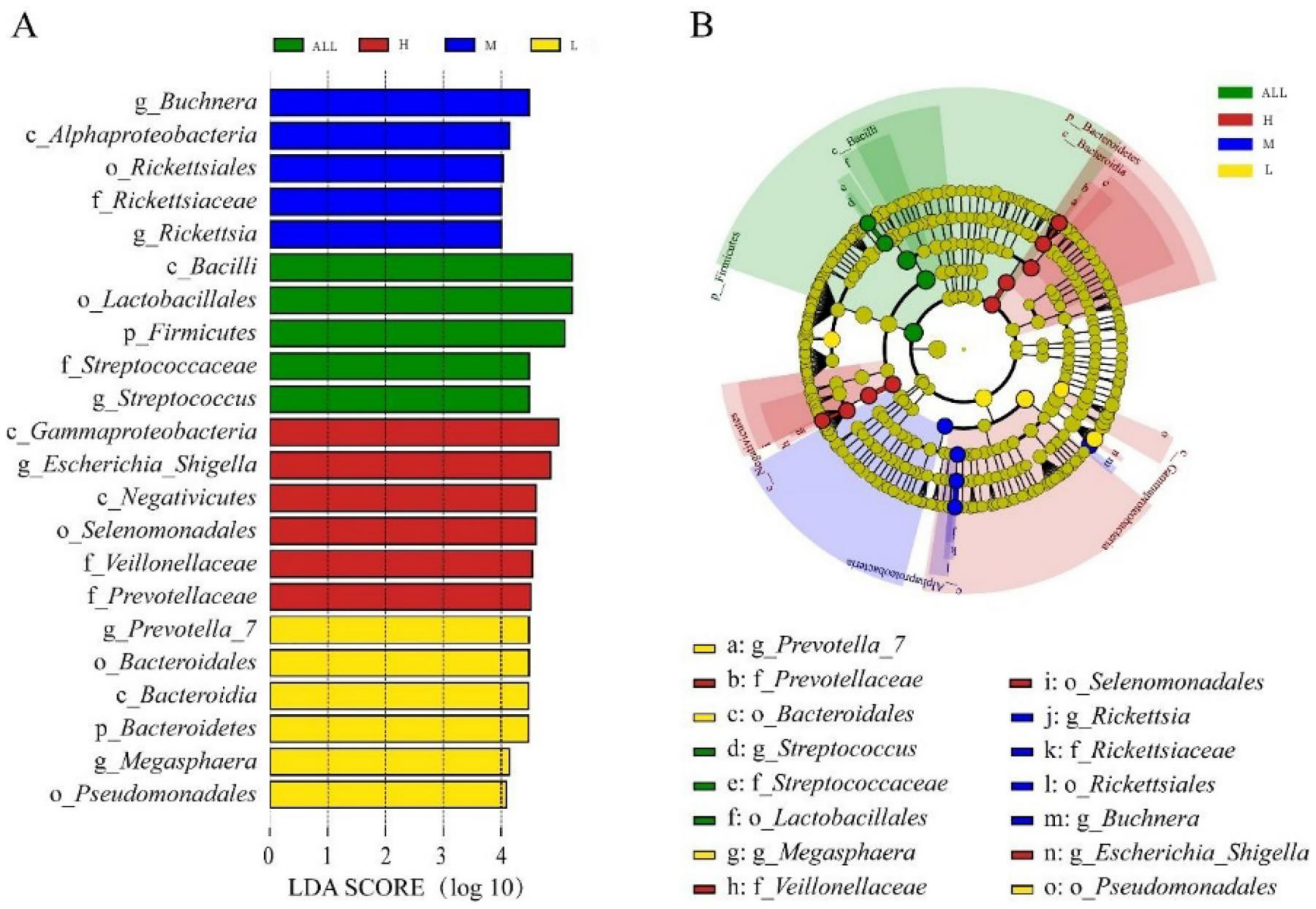
The Linear discriminant analysis effect size (LEfSe) method identifies the significantly different abundant taxa of bacteria.

**Figure 6.**
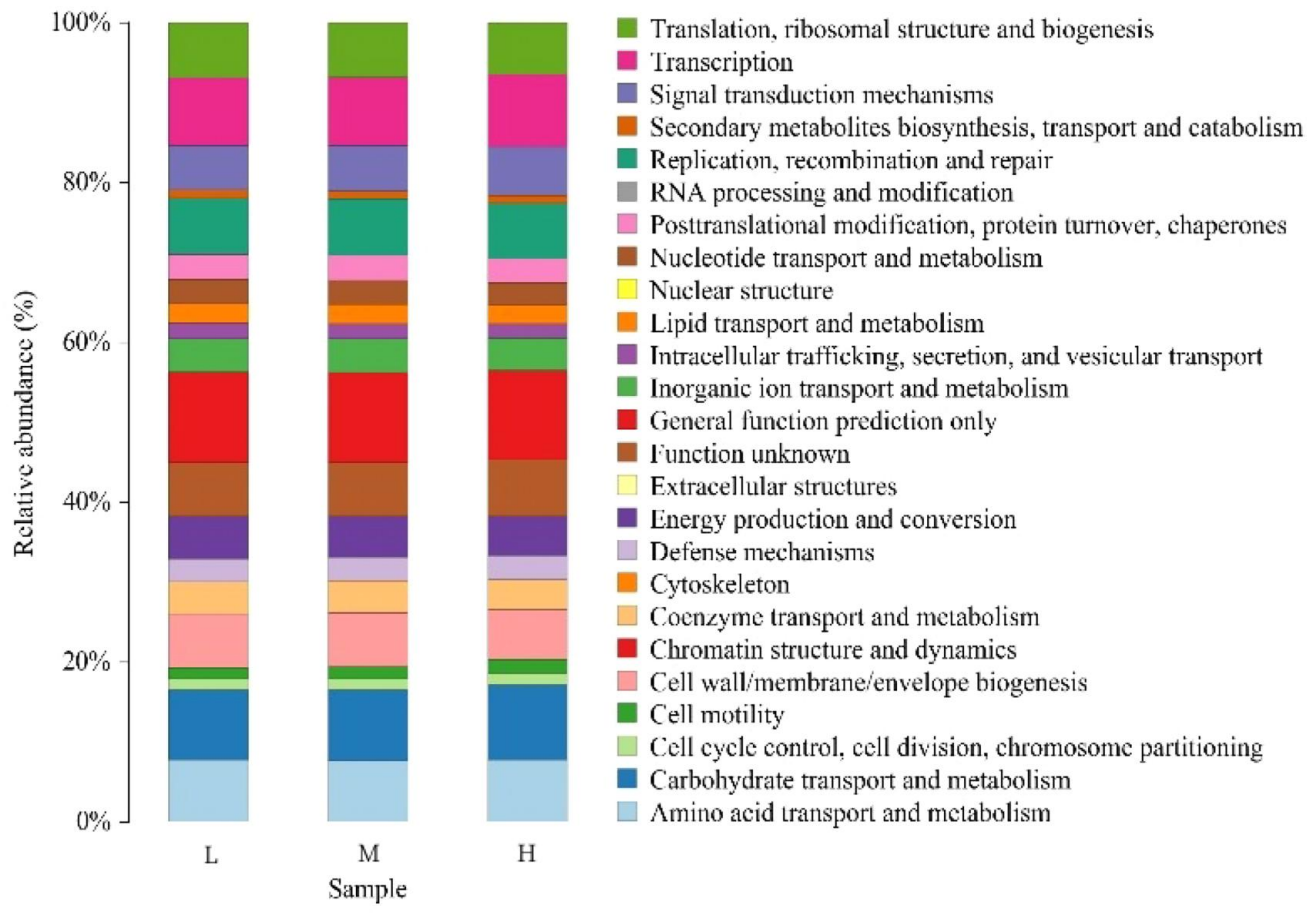
Ruminal fluid samples microbiota KEGG analysis.

We performed LEfSe analyses to identify the significance of different taxa (relative abundance >1%) among all groups, and LDA results from the LEfSe analysis were showed in Figure 5. In H group, Gammaproteobacteria, Escherichia_Shigella, Negativicutes, Selenomonadales, Veillonellaceae and Prevotellaceae were significantly abundant taxa. In M group, Buchnera, Alphaproteobacteria, Rickettsiales, Rickettsiaceae and Rickettsia were significantly abundant taxa. In L group, Prevotella_7, Bacteroidales, Bacteroidia, Bacteroidetes, Megasphaera and Pseudomonadales were significantly abundant taxa. To investigate the functional capacity of the ruminal fluid microbiota communities, PICRUSt was used to further analysis the KEGG pathway compositions (Fig. 6). The results showed that the second level KEGG pathway of Amino acid transport and metabolism, Carbohydrate transport and metabolism, Cell cycle control, Cell motility, Cytoskeleton, Energy production and metabolism were enriched. The ruminal fluid microbiota of alfalfa supplementary feeding showed low immune pathway and high carbohydrate metabolism pathway.

## Discussion

Rumen is an important part of the digestion, absorption, and metabolism of nutrients, and acts as an important barrier against the harmful substances, thereby, playing an important role in the sheep health.^14^ The ruminant need intake 30%–70% dietary roughage to maintain normal rumen function and gastrointestinal microflora environment.^23^ Many researchers have reported that additional hay feeds can elicit a response in terms of reduced weight loss, increased growth and production in grazing sheep farming system.^24^ The sheep supplemented with Alfalfa supplementary feed weight maintenance over winter than the other supplementary feed systems have been widely used to balance nutrition of ruminants fed low quality forage-based diets on confinedness.^25^ Roughage concentration increased (from 55% to 85%) in dietary can change in the substrate available for rumen fermentation and improve rumen fermentation and feed energy utilization efficiency.^26^ Our results are consistent with the previous report, in which a higher NFC/NDF ratio in a dietary contained many rapid fermentative carbohydrates and be useful to increase sheep serum chemistry values and daily body weight gain, it was in accordance with earlier reports for grazing sheep with supplemented feeding in dormant season. There is evidence that CHO deficiencies are common in grazing animals. CHO levels were found below 2.0 mmol/L in grazing sheep, and increased CHO activities in present study can be depend on alfalfa supplementary intake, which increased Cholesterol Oxidase, then compensation of the decreased CHO levels.^27^ LDH is an important enzyme in glycolysis, taking together our results associated with carbohydrate metabolism that LDH of higher NFC/NDF ratio in a dietary contained were significantly greater, the reasons for this phenomenon that high ratio Alfalfa supplementary in sheep dietary might be increased carbohydrate metabolism.

Modern supplementary pattern changed the feedstuff residing time in sheep rumen and improved the amount of carbohydrate and microbial fermentation level.^28^ Acetate significantly decreased and propionate and butyrate significantly increased as dietary non-fibrous carbohydrate (NFC) to neutral detergent fiber (NDF) ratio decreased, reflecting changes in the substrate available for rumen fermentation and fiber digestion depression in heifers.^29^ High NFC/NDF dietary linearly reduce acetate concentrations, resulting in the transition from acetic acid to propionic acid of ruminal fermentation and improving feed energy utilization efficiency.^30^ Ruminal pH depends mainly on the balance of VFAs production and buffer secretion, which are used to reflect rumen natural activities status. VFAs originated from carbohydrates via hydrolysis by ruminal microbes. The observed negative relationship between ruminal pH and VFAs concentration in the present study, which indicated that our sheep dietary could maintain the stability of rumen environment, and ensure the degradation of roughage via rumen microorganisms. Ammonia-N is the most important N source for microbial protein synthesis in the rumen. Serum BUN level is associated with protein uptake, alfalfa was rich in protein, BUN activities increased in higher NFC/NDF ratio in dietary contained. In the present study, Ammonia-N, Total-N and Soluble protein-N presented an observed increasing with an increase in dietary alfalfa concent ratio, which was indicated the larger N resources for microbial growth in Alfalfa hay supplementary intake. A higher NFC/NDF ratio dietary many accumulate excessive VFA, and have a greater efficiency of urea recycling, which helps these animals to survive under poor dietary condition of the Sunan Plateau.

Ruminant rumen encompass a large number of complex and diverse microorganisms, which are critical for the body metabolism and the homeostasis of ruminal barrier by regulating the transformation of nutrients uptake.^31^ A number of studies have shown that low NFC/NDF ratio dietary intake ruminant had impaired ruminal barrier function, which enhanced the transferring of toxic metabolic products from the rumen into blood circulation via ruminal microflora dysfunction and microbial translocation, causing an inflammatory response and tissue dysfunction.^32^ Therefore, the dynamic equilibrium of the structure and function of rumen microbiota contributes to explore underlying mechanism of sheep rumen digestive function in alfalfa supplementary feeding system via changing dietary non-fibrous carbohydrate (NFC) to neutral detergent fiber (NDF) ratio.

The 16S rRNA microbial sequencing technology is an important method to explore the functions and activities of the ruminant gastrointestinal microbiota.^33^ In the present study, 16S rRNA gene-based pyrosequencing data highlighted the predominance of Ruminococcus, Ruminococcaceae and Prevotella for all treatments. Although there was no difference between the four treatments, high NFC/NDF ratio dietary treatment increased the relative abundance of Ruminococcus and Ruminococcaceae, and decreased the abundance of Prevotella, which improved plant polysaccharide degradation and supplemented host metabolism. Prevotella plays an important role in the digestion of starch, glycans, protein, and hemicellulose as energy sources, Ruminococcus and Ruminococcaceae belonging to the Firmicutes, are known to degrade celluloses in the rumen.^34^ Ruminant gastrointestinal microorganisms adjusted their own protein synthesis and gene expression to assist in the digestion and synthesis of nutrients.^35^ The abundance of Ruminococcus changed, which led to the changes of fiber digestive ability and the formation of short chain fatty acids (SCFAs) in different NFC/NDF ratio dietary. It is well-known that the SCFAs provides energy, enhance digestion, and regulates the metabolism in ruminal epithelial cells by binding to G protein–coupled receptors of SCFAs. Meanwhile, the ruminal epithelial cells proliferated and differentiated by inhibiting the histone deacetylase.^36^ The expression of low immune pathway and high carbohydrate metabolism pathway genes appeared to be distinguishing features of the high ratio alfalfa-supplementary rumen microorganisms.

## Conclusions

This study confirmed that the fermentation characteristics and ruminal bacterial community composition were altered under different dietary NFC/NDF ratios in Gansu alpine fine-wool sheep. Concentrations of propionate and TVFA were decreased under a dietary NFC/NDF ratio of 1.92. Meanwhile the high NFC/NDF ratio dietary increased the richness and diversity of the rumen microbiota. This study provided a better understanding of the bacterial ecosystem variation in Gansu alpine fine-wool sheep fed the different NFC/NDF dietary.
